# Mitochondria dysfunction in the pathogenesis of Alzheimer’s disease: recent advances

**DOI:** 10.1186/s13024-020-00376-6

**Published:** 2020-05-29

**Authors:** Wenzhang Wang, Fanpeng Zhao, Xiaopin Ma, George Perry, Xiongwei Zhu

**Affiliations:** 1 Department of Pathology, Case Western Reserve University, 2103 Cornell Road, Cleveland, OH 44106 USA; 2 College of Sciences, University of Texas at San Antonio, San Antonio, TX USA

**Keywords:** Alzheimer’s disease, Mitochondrial dysfunction, Bioenergetics, mtDNA, Mitochondrial dynamics, Axonal transport, Mitochondrial biogenesis, Mitochondrial quality control, ER-mitochondria association, Mitochondrial proteostasis

## Abstract

Alzheimer’s disease (AD) is one of the most prevalent neurodegenerative diseases, characterized by impaired cognitive function due to progressive loss of neurons in the brain. Under the microscope, neuronal accumulation of abnormal tau proteins and amyloid plaques are two pathological hallmarks in affected brain regions. Although the detailed mechanism of the pathogenesis of AD is still elusive, a large body of evidence suggests that damaged mitochondria likely play fundamental roles in the pathogenesis of AD. It is believed that a healthy pool of mitochondria not only supports neuronal activity by providing enough energy supply and other related mitochondrial functions to neurons, but also guards neurons by minimizing mitochondrial related oxidative damage. In this regard, exploration of the multitude of mitochondrial mechanisms altered in the pathogenesis of AD constitutes novel promising therapeutic targets for the disease. In this review, we will summarize recent progress that underscores the essential role of mitochondria dysfunction in the pathogenesis of AD and discuss mechanisms underlying mitochondrial dysfunction with a focus on the loss of mitochondrial structural and functional integrity in AD including mitochondrial biogenesis and dynamics, axonal transport, ER-mitochondria interaction, mitophagy and mitochondrial proteostasis.

## Background

Alzheimer’s disease (AD) is one of the most prevalent neurodegenerative diseases in the world [1–3]. The major symptom presents as deterioration of cognition and memory functions due to the progressive and selective loss of neurons in forebrain and other brain areas. The debilitating neurological conditions of the disease cause severe disability in AD patients with progression of the disease. Unfortunately, none of the current therapies cure the disease and all are very limited in their ability to slow down its progression. More importantly, there is increased prevalence and incidence of AD in the world and by 2050, 1 in 85 persons worldwide will be living with the disease and 43% of those afflicted need a high level of care [4, 5]. Considering the huge social and economic burdens devoted to the care of AD patients, enormous effort is focused on exploring effective interventions to alleviate the symptoms or even cure the disease. During the past decades, studies suggested that multiple factors [6] including biological factors (e.g., aging, gender, body weight, etc.), environmental factors (e.g., lifestyle, toxins, brain injury, etc.), and genetic factors (e.g., APP, PS1, and PS2 genetic mutation in familial AD and susceptibility genetic polymorphisms in sporadic cases) contribute to the pathogenesis of AD. Although accumulated knowledge greatly expanded our understanding of AD, the underlying mechanism of AD pathogenesis remains elusive.

Mitochondria are conserved organelles that carry out multiple essential functions in different cellular processes [7]: Many neuronal activities are energy-taxing and human nervous system consumes a great deal of energy, and mitochondria are the major energy source providing ATP through oxidative phosphorylation to maintain the normal neuronal homeostasis and function. Mitochondria are essential for the biosynthesis of essential iron-sulfur center and heme in neurons, and are involved in the presynaptic transmitter synthesis in synapses. Mitochondria provide important buffering machinery to regulate calcium concentration during signal transduction, which is of particular importance to excitable cells such as neurons. Neurons are long-lived cells with the same life span as the organism. As the essential hub in regulating cell survival and death under various stresses, mitochondria safeguard neuronal survival through a variety of stresses during long neuronal lives. In order to perform these various functions, mitochondria are dynamically interacting with one another and with other cellular organelles to coordinate mitochondrial stress response under physiological and pathological conditions. It is therefore not surprising that disturbances of mitochondrial function are closely associated with the mechanisms underlying nervous system abnormalities including neurodegenerative diseases [7].

To date, a large body of research has shown extensive mitochondria abnormalities in the brain of AD patients [8]. Consistent with the observation that impaired energy metabolism invariantly precedes the clinical onset of AD, mitochondrial dysfunction has been established as an early and prominent feature of the disease [8, 9], suggesting a critical role in the pathogenesis of AD. Here we will review evidence of mitochondrial abnormalities in the brain of human AD patients and discuss in more detail recent advances in the understanding of mechanisms underlying mitochondrial dysfunction in AD which may offer novel targets for future therapeutic development.

## Impaired energy metabolism implicates mitochondrial dysfunction in AD

Brain constitutes on average 2% of the total body weight, but utilizes 25% of total body glucose and 20% of body oxygen consumption in resting awake state. As one of the high-energy consuming organs, brain is vulnerable to impaired energy metabolism such that even mild changes in energy metabolism in human brain closely associates with the disturbance in nervous function. In fact, impaired energy metabolism is one of the earliest and most consistent features in AD.

Glucose is the predominant substrate for the human adult brain under physiological conditions, and its utilization is widely used as one primary measure to assess energy metabolism in the brain. A large body of evidence demonstrated significantly reduced glucose utilization as an early and consistent feature in AD, which actually occurs decades before the onset of disease [10–13]. Using fluoro-2-deoxyglucose positron-emission tomography (FDG-PET), greater decline in glucose utilization was consistently found in the hippocampus and cortex in AD brain as compared to individuals without dementia. Among many brain regions, the posterior cingulate cortex is metabolically affected in the earliest clinical stages of AD [10]. Glucose hypometabolism, to a lesser extent in terms of magnitude or spatial distribution, was also observed in patients with mild cognitive impairment (MCI), a prodromal stage of AD, suggesting an early role in the course of disease [12]. An 84-months longitudinal study clearly demonstrated an ApoE4-associated brain-region specific longitudinally declined glucose metabolism pattern in the context of MCI [14]. Such an early role is further supported by the finding of abnormally low rates of glucose metabolism in the vulnerable brain regions in young adults carrying apoE4 allele in their 20s, several decades before the possible onset of dementia [15]. The extent and topography of glucose hypometabolism correlated with symptom severity and also reflected the regional distribution of impaired synaptic activity and density in AD [16, 17]. Accepted as a hallmark of the disease, cerebral glucose hypometabolism assessed with FDG-PET is now used as a common biomarker for early detection of AD and can predict the conversion from MCI to AD with reasonable sensitivity and accuracy [18, 19], which underscores the critical role of impaired energy metabolism in the course of AD.

Multimodal imaging studies, using both FDG PET and amyloid PET biomarkers, have investigated the relationship between amyloid plaque deposition and glucose metabolism. In autosomal-dominant AD mutations carriers, longitudinal Aβ depositions increase in nearly every cortical region 15–25 years before the estimated age of onset, followed by reduced glucose metabolism in selective cortical region approximately 5–10 years later (i.e., amyloid-first biomarker profile pathway), suggesting that glucose hypometabolism could be a secondary event after Aβ depositions in AD pathogenesis in these cases [13, 20, 21]. Although reductions in regional glucose metabolism are associated with global amyloid pathology, there is poor association between regional amyloid pathology and regional hypometabolism when they are compared side-by-side in the same subjects: only 1 out of 404 regions of interest showed a negative association between amyloid plaque deposition and glucose metabolism [22]. This study suggests that glucose hypometabolism, even as a secondary event in the cases of autosomal-dominant AD, may play an essential role in the ensuing clinical onset of the disease. Repeated failure of Aβ-centered clinical trials suggests that it may be too late to target Aβ in AD patients or even MCI patients when some toxic cascade of events have been initiated after adecade of presence of amyloid pathology. These critical secondary pathogenic events such as impaired energy metabolism may provide an extended time window for therapeutic intervention. Therefore, it is still of paramount significance to understand the essential role and mechanisms underlying impaired energy metabolism in AD, even as a secondary event.

On the other hand, energy hypometabolism may play a primary role in a subset of sporadic AD patients. In a population study, while the majority (60%) incident amyloid-positive subjects followed the amyloid-first biomarker profile pathway, 27% had an abnormal FDG-PET at baseline, suggesting the existence of “hypometabolism-first” biomarker profile pathway to preclinical AD [23]. FDG PET studies have identified AD-like glucose hypometabolism in ApoE4 carriers without amyloid deposition who probably also followed this hypometabolism-first biomarker profile pathway since these carriers are likely to develop amyloid pathology in the future [24, 25]. While it is possible that Aβ dysmetabolism prior to amyloid plaque formation or other pathophysiologies such as tau or TDP43 may underlie glucose hypometabolism in these cases, the hypometabolism-first biomarker profile pathway to preclinical AD lends strong support to a primary role for energy hypometabolism in the pathogenesis of at least a subset of sporadic AD patients.

Glucose metabolism is a multi-step process involving glucose transportation and intracellular glucose metabolism. Abnormalities involving almost all of these steps from glucose transportation abnormalities including insulin resistance, abnormal glucose transporter [26] and/or blood flow [27], to intracellular glucose metabolism disturbance including abnormalities in cytosolic processes (i.e., glycolysis and pentose phosphate pathway), in addition to abnormal mitochondria-dependent processes (TCA cycle and oxidative phosphorylation as discussed in details in the next section), were identified in AD brain, which all could contribute to glucose hypometabolism. It is perhaps not impossible that one or more of these factors may play a more important role than other factors in causing cerebral glucose hypometabolism in AD, but it would be challenging to identify such major player(s) given the complex interactions among these factors and the possibility that different AD variants may have differences in the major factor(s) involved.

Nevertheless, glucose hypometabolism in AD brain was generally interpreted as impaired energy metabolism through oxidative phosphorylation, which thus strongly implicates the involvement of mitochondrial dysfunction early in the course of AD. Along this line, glucose hypometabolism in frontal, temporal, and parietal cortices of patients with AD were closely correlated with the reduced levels of blood thiamine diphosphate (TDP), a critical coenzyme of pyruvate dehydrogenase (PDHC) and α-ketoglutarate dehydrogenase (KGDHC) in the Krebs cycle and transketolase in the pentose phosphate pathway [28]. Similar to glucose hypometabolism, TDP reduction and reduced activities of thiamine-dependent enzymes are also a significant and common feature in patients with AD [29, 30]. Oxygen metabolism measured by positron-emission tomography (PET) detection of Oxygen-15 is another primary measure for brain energy metabolism, which provides direct evidence for mitochondrial function through electron transport chain (ETC) in the brain. Cerebral metabolic rate of oxygen was significantly decreased in the frontal, parietal and temporal cortex in AD, which showed significant correlation with severity of dementia [31–33]. Another study found significant correlation between reduced oxygen metabolism and electroencephalogram slowing in the parieto-temporal regions of AD brain [34]. In fact, multiple lines of evidence demonstrated impaired bioenergetics machinery, especially the tricarboxylic acid (TCA) cycle and the ETC chain, in the mitochondria in AD. Collectively, these studies strongly suggest that mitochondrial dysfunction likely plays a critical role in glucose hypometabolism and energy impairment in AD.

## Mitochondrial deficits in AD

### Disrupted mitochondrial bioenergetics in AD

Consistent with impaired energy metabolism in AD, gene expression studies repeatedly identified defects in mitochondrial related metabolic pathways in AD, which provided direct evidence for impaired bioenergetic machinery in mitochondria of AD. For example, a genome-wide transcriptome study in laser-capture micro-dissected neurons found significantly greater proportion of underexpressed nuclear genes encoding mitochondrial ETC subunits in the posterior cingulate cortex than those in the primary visual cortex, a region that is relatively spared metabolically in AD vs. control [35]. A microarray analysis and quantitative RT-PCR studies found 15 out of 51 members of the glycolytic, TCA cycle, oxidative phosphorylation, and associated pathways were significantly downregulated in AD [36]. More recent microarray data confirmed significant downregulation in nuclear-encoded but not mitochondria-encoded OXPHOS genes in the hippocampus of AD patients, which however was puzzlingly increased in the hippocampus from MCI patients [37]. Complex I of OXPHOS was downregulated while complexes III and IV showed increased mRNA expressions in both early and definite AD brain specimens [38]. A bioinformatics analysis of four transcriptome datasets for the hippocampus of AD patients identified OXPHOS pathway as one of most significant pathways involved in AD [39]. Gene set enrichment analysis demonstrated that mitochondrial oxidative phosphorylation (OXPHOS) downregulation and mitochondrial import pathways disruption were hallmarks of AD [40].

Proteomic and protein expression studies also confirmed underexpressed proteins in OXPHOS pathway as one of the most affected processes in the cortex of AD patients [41]. Quantitative proteomics approaches revealed differentially altered mitochondriomes in AD brain are different from aging-associated changes suggesting that dysregulated mitochondrial complexes (i.e., ETC complexes and ATP-synthase) are the potential driver for pathology of the AD [42]. Significantly decreased immunocytochemical staining of various complexes including Complex I and IV in various brain regions in AD was also reported [43, 44]. Given that the activity of some enzymes is regulated by posttranslational modification, it would be of importance to determine the alterations in the enzymatic activities of these enzymes in AD brain. The activity of enzymes involved in TCA cycle changed in AD following a distinct pattern: the dehydrogenases/decarboxylases (including PDHC, ICDH and KGDHC) were reduced while dehydrogenases (including SDH and MDH) were increased and all of these changes correlated with the clinical state [45]. Biochemical studies of enzyme activities in mitochondria isolated from autopsied AD brain demonstrated a generalized depression of activities of all ETC complexes with most dramatic reduction in COX activity [46]. Some studies reported more specific defect in the COX activity [47–49]. For example, careful histochemical quantification of cytochrome c oxidase activity in the metabolically affected posterior cingulate cortex and less affected primary motor cortex between AD and age-matched control revealed significantly lower COX activity in the former but not in the latter [43]. More recent studies demonstrated that mitochondrial ATP synthase activity is impaired in the brain of AD patients due to loss of oligomycin sensitive conferring protein subunit [50] and/or changes in the O-GlcNAcylation of ATP synthase subunit α [51].

However, contradictory results were reported where several groups failed to find difference or even increased expression of OXPHOS genes in AD compared to control, some of which may be attributed to different brain regions and/or the widespread heterogeneity of sporadic AD brain samples used. For example, decreased expression of complexes I, II, IV and V was found in the entorhinal cortex but not in the frontal cortex in AD Braak stages V-VI compared with stages I-II [52]. Cell-type specific effects may also contribute to the variance as evidenced by a recent study demonstrating significantly reduced nuclear-encoded OXPHOS genes in laser-captured hippocampal pyramidal neurons from AD compared to that of control cases despite increased expression of these genes in the whole homogenates from these same cases [53].

Overall, these gene and protein expression studies along with biochemical studies clearly demonstrated extensive mitochondria bioenergetics defects in AD brains, which makes it a valuable therapeutic target. It remains to pinpoint specific alterations in oxidative metabolism and to generalize the role of mitochondrial dysfunction in regional vulnerability and pathogenesis of AD, which requires consistent measurement of these parameters across different brain regions. Furthermore, it has been demonstrated that reductions of activity of complex I, III and IV that reach certain thresholds such as greater than 70% in some studies were necessary to produce a significant decrease in ATP production [54, 55]. It thus remains unresolved whether and how chronic but mild impairment in the ETC complexes (i.e., around 15–50% reduction in complex I or IV [8]) is sufficient to cause bioenergetics impairment seen in AD brain. Nevertheless, it should be noted that such threshold appears widely varied (for example, a threshold of 25% [56], 35% [55] or 70% reduction [54] for complex I were suggested by different studies) which could be tissue specific and influenced by other factors such as antioxidant status. For example, glutathione depletion eliminates the complex I threshold in PC12 cells [56]. A combination of mild impairments in different individual complexes may also effectively lower the threshold to produce bioenergetic dysfunction.

### Increased oxidative stress in AD

Reactive oxygen species (ROS) are unavoidable byproducts during electron transport of aerobic respiration in the mitochondria due to electron leaks at complex I and complex III and it is estimated that mitochondria contribute approximately 90% of the cellular ROS [57]. While ROS serve important signaling roles, when in excess, they lead to oxidative stress with extensive damage. Mitochondria are susceptible to oxidative damage despite the presence of an antioxidant system and damaged mitochondria are less efficient producers of ATP and more efficient producers of ROS. Therefore, increased oxidative stress could be both the cause and consequence of mitochondrial dysfunction.

A large body of evidence demonstrated increased oxidative damage to almost all types of macromolecules in the brain of AD patients including proteins, sugar, lipid and nucleic acids [58]. For example, significant increase in protein carbonyls and 3-nitrotyrosine modification as protein oxidation markers and elevated glycation and glycooxidation marking oxidative modifications to sugars were widely reported in the brains of patients with AD and MCI [9]. Lipid peroxidation products such as reactive aldehydes including 4-hydroxynonal, malondialdehyde (MDA), and acrolein were increased in multiple brain regions affected in AD and MCI [59]. AD brains demonstrated significant increase of 8-hydroxydeoxyguanosine (8-OHdG) and 8- hydroxyguanosine (8-OHG) respectively in DNA (including mtDNA, which will be discussed in more detail in the next session) and RNA [60]. On the other hand, significantly decreased antioxidant levels and/or changes in the expression and activities of antioxidant enzymes were also reported in AD brain. Recent in vivo imaging studies confirmed AD-dependent reduction of glutathione levels in affected brain regions in AD and MCI patients [61, 62], which strongly correlated with declines in cognitive function. The increase in oxidative stress markers strongly associated with significant loss of synaptic proteins in the brains of MCI and pre-AD patients too [63]. Detailed analysis of stable oxidation modifications such as lipid peroxidation and protein glycation revealed widely distributed cumulative oxidative damage in neurons both with and without AD-associated pathology; interestingly, short-lived oxidative damage such as oxidized DNA/RNA and 3-nitrosylation was prominent in neurons without pathology but reduced in cells with pathology [9]. These studies suggest that oxidative stress occurs earlier than the formation of AD-related pathology and AD-associated pathology could play a protective role in fighting against ROS production/damage.

Redox proteomics studies to identify oxidatively modified proteins contributed a great deal to the understanding of the diseased proteome in the various stages of AD and shed light on potential molecular pathways involved [58]. These studies found that many antioxidant enzymes were oxidized which likely compromised their functions and contributed to increased oxidative stress in AD. For example, glutathione-S-transferase Mu, peroxiredoxin 6, multidrug-resistant protein 1 or 3, and GSH were all found to be HNE-modified and/or nitrated in various brain regions of MCI and AD patients [64]. This unbiased approach also revealed that many proteins involved in the energy metabolic processes were posttranslationally modified either by lipid conjugation or by reaction with ROS in the brains from patients with AD or MCI. For example, ATP synthase, the enzyme responsible for the final step of ATP production, was found to be HNE-modified and nitrated in AD and MCI hippocampus [64]. HNE-modification or carbonylation was found in aconitase in the TCA cycle and creatine kinase in ATP maintenance [64]. These data suggest that increased oxidative stress contributed to mitochondrial dysfunction and impaired energy metabolism in AD.

### Disturbed mitochondrial genomic homeostasis in AD

Mitochondria maintain their own DNA called mtDNA, which is a multicopy (1–10 copies per mitochondrion), extrachromosomal genome that codes for 13 mitochondrial core proteins of the ETC complexes and 2 rRNA and 22 tRNAs necessary for mitochondrial protein synthesis [65, 66]. While mtDNA is critical to the proper function of mitochondria, it is prone to oxidative damage due to its proximity to the site of ROS generation and relative lack of DNA-protective histones and efficient DNA repair mechanisms, which gives rise to mutations [67, 68]. Mutations in mtDNA, whether through inheritance or gradual somatic accumulation, propagate through clonal expansion, which may eventually gain momentous deleterious effects after exceeding critical threshold, and compromise mitochondrial function and result in cell death and disease [8].

Many patients with primary mtDNA mutations demonstrated pronounced cognitive deficits quite similar to those commonly seen in AD [69], which supports a critical role of mtDNA in proper cognitive function. Interestingly, it is reported that in families with a history of dementia, a consistently identified risk factor for AD, maternal transmission is significantly more frequent than paternal transmission. Along this line, maternal family history of AD is associated with increased atrophy in AD-vulnerable brain regions [70], a pattern of progressive reduction of brain glucose metabolism [71, 72] and higher white matter hyperintensity load in temporal and occipital lobes [73] in cognitively normal individuals, pointing towards family of origin effects. Given the maternal inheritance of mtDNA, this implicated a potential role of inherited mtDNA variability in AD. Indeed, while no primary mtDNA mutations were associated with AD, multiple studies have found that mtDNA SNPs and germline variants (i.e., haplogroups) likely play a role in AD (Table 1): for example, haplogroup UK is associated with higher risk of AD while haplogroup T is protective [74, 75]. Interaction between mtDNA inherited variability and other factors such as gender or apoE alleles may change the susceptibility to AD: some mtDNA haplogroups (K and U) seem to neutralize the harmful effect of the apoE4 allele [76]; haplogroup U is associated with higher risk for men but reduced risk for women [77]. However, an association between mtDNA inherited variability and the development of AD remains inconclusive at this time since a large-scale mtDNA haplogroup study from three Caucasian populations failed to replicate these findings [78]. Interestingly, this study noticed some evidence for association between individual mtDNA SNPs previously implicated in AD within subsets of samples, dependent on geographic locations, suggesting that geographic difference in the fine details of the sub-haplogroup structure of mtDNA could contribute to the inconsistency between studies.

Involvement of somatic mtDNA mutations were also extensively studied in AD with a focus on the common 5-kb deletion (mtDNA Δ4977) occurring between positions 8470–8482 and 13,447–13,459, which presumably affects the expression of ETC complex I, III and V in AD. Earlier quantitative PCR studies found age-related accumulation of this deletion in frontal cortex [79] and a striking 15 fold increase of this deletion in AD patients younger than 75 years of age [80]. A comprehensive assessment of mtDNA rearrangement events found significantly higher levels F-type and R-type rearrangements, in addition to deletion, in AD brain [81]. AD brains had an average 63% increase in heteroplasmic mtDNA point mutations in the control-region (CR) and certain AD brains harbored the disease-specific CR mutations at levels up to 70–80% heteroplasmy, which preferentially altered regulatory elements of known mtDNA and suppressed transcription and replication of mtDNA [82].

However, conflicting results were reported since some groups found no changes in the aggregate burden of brain mtDNA point mutations between AD and control [83–85], which was probably owing to the small sample size and approach difference [86]. Lack of distinction of cell-specific mtDNA may also contribute to the variability since more sensitive studies by in situ hybridization [87] and laser capture microdissection in single hippocampus neurons or glial cells followed by a multiplex real-time qPCR method [88, 89] demonstrated increased neuronal but not glial occurrence of mtDNA Δ4977 in AD. More detailed study revealed markedly increased ratio of mtDNA Δ4977 over normal mtDNA in COX negative neurons that were selectively enriched in AD [90]. Different disease stage may also contribute to the variability. A recent study on enriched neuronal mtDNA using more accurate next generation sequencing methodology, which eliminates sequencing errors associated with PCR and DNA damage, revealed significantly elevated frequency of mtDNA point mutation in the hippocampus of patients with early stage AD (i.e., individuals who were not demented, but had high Braak staging characteristic of AD dementia), but not in patients with pathologically confirmed AD dementia [91]. This study not only suggested an early role of mtDNA mutations in AD, but also demonstrated that mutated mtDNA may be lost as neurons die when disease progresses. It also needs to be noted that ancient accumulated polymorphisms and somatic mutations are not mutually exclusive but their combined effects are not studied.

It was believed that increased mtDNA mutations are due to increased oxidative damage found in AD brain [8]. Indeed, mtDNA had approximately 10-fold higher levels of oxidized bases than nuclear DNA and mtDNA underwent an average of threefold increase in oxidative damage in the brain from AD patients compared to age-matched controls [92, 93]. In fact, levels of oxidized nucleic acids in mtDNA were found to be significantly elevated in preclinical Alzheimer’s disease (PCAD) and MCI patients [94], suggesting that this is an early event during the course of disease. More recent studies demonstrated decreased OGG1 activity [95] and impaired base-excision repair (BER) activity in both AD and MCI patients [83], suggesting significant contribution of replication error to increased mtDNA mutations in AD [91]. Other modifications to mtDNA may also impact its transcription and function. For example, increased 5-methylcytosine levels are found in the D-loop region of mtDNA in brain samples with AD-related pathology [96]. On the contrary, there is a decreased methylation of the D-loop region in peripheral blood mtDNA from LOAD patients [97]. The implication of these findings to human AD pathogenesis remains to be explored.

Overall, these studies suggest a likely critical role of mtDNA variabilities, mutations and modification in the pathogenesis of AD. Indeed, it has been proposed by the mitochondrial cascade hypothesis that inherited mtDNA variants determine one’s vulnerability and the accumulation of brain somatic mtDNA modifications and mutations reflecting the influence of the environment along aging determines the manifestation of the phenotype [8]. However, specific mtDNA alterations, if any, and a potential causal role of mtDNA alterations in AD pathogenesis are yet to be proven. Additionally, mtDNA alterations are found in other neurodegenerative diseases but not specific to AD, and how they specifically relate to AD-type changes await further exploration.

## Mechanisms underlying mitochondrial dysfunction in AD

**Fig. 1:**
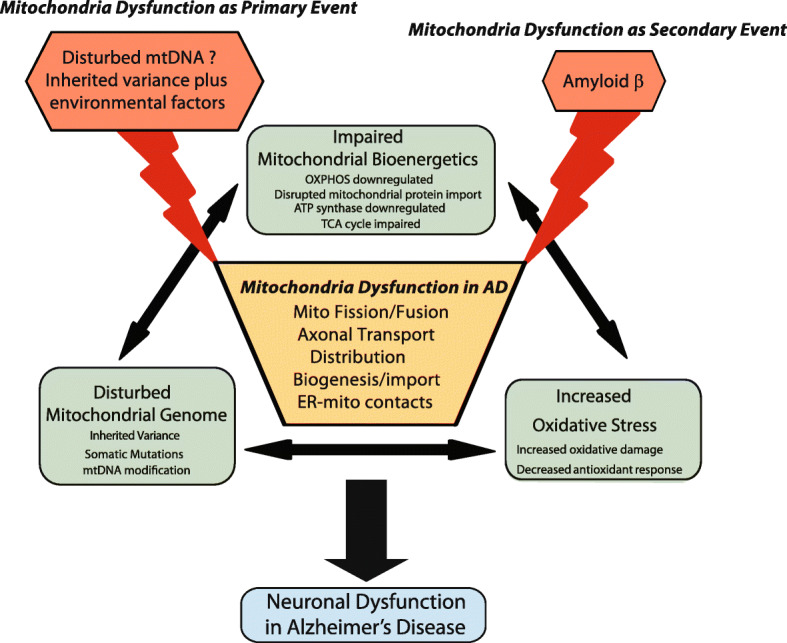
Critical role of mitochondrial dysfunction in AD. Mitochondrial dysfunction plays a critical role in AD either as a primary or secondary event. In either case, impaired mitochondrial bioenergetics, increased oxidative stress and disturbed mitochondrial genome are consistent features of mitochondrial abnormalities in AD, all which interact with each other to form a deleterious downward spiral. While the relative importance of these abnormalities in triggering mitochondrial dysfunction may vary among patients with AD depending on the unique biological, environmental and genetic factors of each individual, any of these abnormalities could induce the other two to complete the downward spiral to mediate and amplify neuronal dysfunction and neurodegeneration. Recent studies revealed mechanisms underlying the loss of integrity of mitochondria, which provides mechanistic link among these abnormalities and offers multiple novel intervention sites to improve mitochondrial function in AD

The existence of hypometabolism-first biomarker profile pathway to preclinical AD along with the extensive evidence that mitochondrial abnormalities could lead to AD-related deficits in model organisms suggest that mitochondrial dysfunction could play a primary role at least in a subset of sporadic AD patients (Fig. 1). As to the autosome dominant AD mutations carriers where amyloid-first biomarker profile pathway to AD plays the primary role, impaired energy metabolism is an invariant feature preceding clinical onset of the disease suggesting that mitochondrial dysfunction likely plays an upstream role, although secondary to other fundamental AD events, in mediating and amplifying neuronal dysfunction and neurodegeneration in AD (Fig. 1). Regardless whether mitochondrial dysfunction plays a primary or secondary role, impaired mitochondrial bioenergetics, increased oxidative stress and disturbed mitochondrial genome are consistent features of mitochondrial abnormalities in AD, all which interact with each other to form a deleterious downward spiral. While the relative importance of these abnormalities in triggering mitochondrial dysfunction may vary among patients with AD depending on the unique biological, environmental and genetic factors of each individual, any of these abnormalities could induce the other two to complete the downward spiral to mediate and amplify neuronal dysfunction and neurodegeneration (Fig. 1). Considering the essential role mitochondrial dysfunction plays in the pathogenesis of AD, the mechanisms underlying mitochondrial impairments and related neuronal loss in AD have been extensively studied, which generated novel insights that may offer new targets for future therapeutic development.

**Table 1:** mtDNA changes in AD

Mutation type	Affected mtDNA region	Analysis Method	Changes in AD	Reference
mtDNA Δ4977	I, III and V	PCR	Increased	Corral-Debrinski et al. [80]
mtDNA Δ4977	I, III and V	PCR	No changes	Blanchard et al. [79]
mtDNA Δ4977	I, III and V	In situ hybridization	Increased	Hirai et al. [87]
mtDNA Δ4977	I, III and V	PCR	No changes	Bender et al. [90]
mtDNA Δ4977	I, III and V	PCR	Increased	Krishnan et al. [88]
mtDNA Δ4977	I, III and V	Realtime PCR	No changes	Strobel et al. [89]
DNA Rearrangement	Mitochondrial genome	Next generation sequencing	Increased	Chen et al. [81]
Point mutation	D-loop region	PCR/Sanger sequencing	Increased	Coskun et al. [82]
Point mutation	Mitochondrial genome	Random mutation capture	No changes	Soltys et al. [83]
Point mutation	Mitochondrial genome	PCR-cloning-sequencing	Increased	Lin et al. [84]
Point mutation	Mitochondrial genome	Next generation sequencing	Increased	Hoekstra et al. [91]
DNA methylation	D-loop region	TaqMan PCR	Increased	Blanch et al. [96]
DNA methylation	D-loop region	Realtime PCR	Decreased	Stoccoro et al. [97]

### Abnormal mitochondrial fusion and fission in AD

**Fig. 2:**
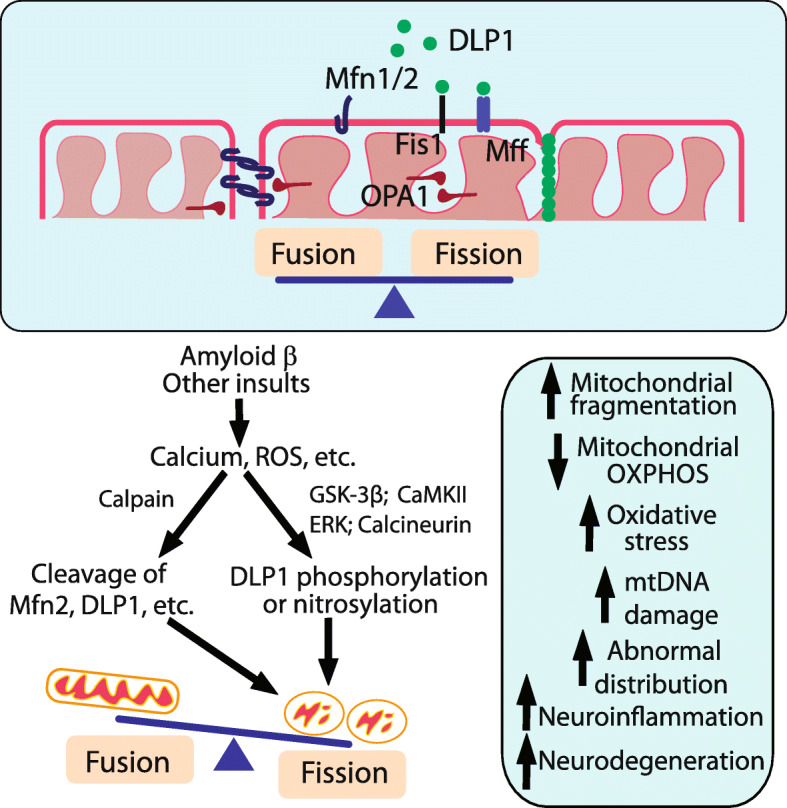
Abnormal mitochondrial fusion and fission in AD. (Top) A family of large GTPases regulate balanced mitochondrial fusion (e.g. Mfn1/2, OPA1) and fission (DLP1). For mitochondrial fission, mitochondrial outer membrane proteins (e.g. Fis1, Mff and others) recruit cytosolic DLP1 protein to mitochondria that oligomerize and form a ring structure around fission site. (Bottom) Amyloid-β and other AD related insults cause neuronal calcium influx and increased ROS production that activate downstream proteases (calpain) and protein kinase that act on mitochondrial fission/fusion GTPases and disturb mitochondrial fusion and fission in AD

Mitochondria are highly dynamic organelles undergoing continuous fusion and fission in the cytoplasm, a process that is essential for maintaining a healthy pool of mitochondria with proper distribution [98]. The molecular mechanisms of the fusion and fission are still under intensive exploration but accumulating evidence suggest that a group of large GTPase domain-containing proteins play critical roles, which either enhance mitochondrial fission such as DLP1(also referred to as Drp1), or promote mitochondrial fusion such as Mfn1, Mfn2 and OPA1 [99] (Fig. 2). Deficits in either fission or fusion cause human neurological disorders, which underscores the importance of balance of mitochondrial fission and fusion in neuronal function and brain health [99].

Early studies demonstrated ultrastructural damage to the susceptible pyramidal neurons in the biopsied brain tissues of AD [87]. More detailed analysis revealed altered size and number and reduced aspect ratio of mitochondria in these neurons, suggestive of a potential fragmented mitochondrial network in AD brain [100, 101]. Fragmented mitochondria could cause mitochondrial bioenergetics deficits either through negative impact to the proper complex assembly critical for ETC function [102, 103] or enhance ROS generation [104] (Fig. 2). Moreover, it also caused reduced exchange of mitochondrial contents exacerbating mtDNA deficits [99], all prominent features found in AD brain (Fig. 2).

Indeed, biochemical evaluation of AD brains demonstrated significantly reduced protein expression levels of all the large dynamin-related GTPases involved in fission and fusion including DLP1, OPA1, Mfn1, and Mfn2 along with significantly increased levels of fission factor, Fis1, in AD brain [101, 105]. Given that DLP1 and Mfn2 are substrates of calpain and cleaved by calpain activation induced by multiple AD-relevant insults in vitro [106], the fact that reduced levels of these GTPases correlated with calpain activation in AD brain suggests that calpain-mediated degradation could at least contribute to the reduction of these fission/fusion GTPases in AD [106]. Despite some controversial reports on the expression of DLP1 in the AD brain [105], studies from multiple groups demonstrated significant changes in the post-translational modifications of DLP1 consistent with its increased translocation to mitochondria in AD: Cho et al. reported that Aβ induced S-nitrosylation of DLP1 (forming SNO-Drp1) triggered mitochondrial fission, synaptic loss, and neuronal damage in AD [107]. Wang et al. found significantly increased phosphorylation at the Ser616 sites along with increased S-nitrosylation of DLP1 associated with increased mitochondrial DLP1 in AD brain [101]. Interestingly, Manczack et al. reported interactions between oligomeric Aβ and DLP1 as well as hyperphosphorylated tau and DLP1 in AD brain [105, 108]. While it was suggested that these abnormal interactions likely facilitate mitochondrial fragmentation, it remains to be solved where these interactions occur and how they enhance fission activity of DLP1.

Impaired mitochondrial fusion and fission balance having an essential role in mitochondrial dysfunction and pathogenesis of AD was corroborated by studies in both in vitro and in vivo experimental models of AD [9, 87, 100, 101, 109]. Overexpression of wild type or mutant APP caused mitochondrial fragmentation in M17 neuroblastoma cells and primary neurons, which was blocked by beta-APP cleaving enzyme (BACE1) inhibitor, suggesting that Aβ induced this effect [100, 101]. Indeed, exposure to soluble Aβ oligomers caused time- and dose-dependent changes in the expression of mitochondrial fission and fusion proteins along with significant mitochondrial fragmentation and dysfunction [101, 110]. Importantly, inhibition of mitochondrial fission rescued APP- or Aβ-induced mitochondrial deficits and neuronal deficits [100, 101], which established a critical role of mitochondrial dynamics in these models. It has been suggested that calcium signaling-dependent and/or oxidative stress-induced posttranslational modifications to DLP1 mediate Aβ-induced mitochondrial fragmentation: Aβ-induced calcium influx led to increased DLP1 phosphorylation at Ser616 sites through CaMKII-dependent Akt activation that stimulate DLP1 translocation to mitochondria and fission activity [111]. Increased oxidative stress induced by Aβ activates ERK, which in turn leads to DLP1 phosphorylation and mediates downstream toxic effects on mitochondria and neurons [112]. An artificial polypeptide, TAT-Drp1-SpS, could specifically block GSK3β-induced Drp1 phosphorylation and rescue Aβ toxicity related mitochondrial dysfunction in vivo and in vitro [113]. Aβ induced increased S-nitrosylation of DLP1 at Cys644 through enhanced nitric oxide production also enhanced its dimerization and fission activity [107] although this notion was challenged by a later study [114].

Aβ induced abnormal mitochondrial dynamics is an early event during neurodegeneration in vivo in Drosophila models [115, 116]. A 3D electron microscopy study demonstrated a peculiar “beads-on-the-string” morphology of mitochondria in the pyramidal neurons in two different APP transgenic mouse models [117], which likely represents an excessive fission process that is stalled at the last step. Indeed, an in vivo multiphoton imaging study confirmed fragmented mitochondria near amyloid plaques in vivo in an APP transgenic model [118]. Wang et al. further reported mitochondrial fragmentation and ultrastructural damage in the brain of CRND8 APP transgenic mice by both confocal microscopy and electron microscopy studies, accompanying mitochondrial functional deficits at 3 months of age, well before any noticeable amyloid deposition, which suggest that mitochondrial dynamic abnormalities occur early in the course of AD-related changes [109]. Collectively, these studies suggest a tipped mitochondrial dynamics balance towards excessive fission in vivo in various AD mouse models, but falls short in demonstrating a causal role of mitochondrial dynamic abnormalities in neurodegeneration in vivo. Notably, recent studies reported that mitochondrial fragmentation in the cortex and hippocampus caused by unopposed fission due to Mfn2 knockout led to mitochondrial ultrastructural damage and functional deficits along with extensive oxidative stress followed by dramatic neuroinflammation that eventually caused significant neuronal loss [119, 120]. This study demonstrated that mitochondrial fragmentation could initiate a degenerating cascade culminating neurodegeneration that replicates many of the pathological features during the course of AD, thus establishing a causal role of mitochondrial dynamic abnormalities in vivo.

These studies pave the road to pursue abnormal mitochondrial fission as a potential therapeutic target for AD, and so far specific attention focused on the extensively studied mitochondrial dynamic protein DLP1 which adds more evidence of a critical role of mitochondrial dynamics in the course of AD. Chemical inhibition of mitochondrial fission by DLP1 specific inhibitor Mdivi-1 and genetic reduction of DLP1 proteins were explored in AD models in vivo and in vitro. By inhibition of ERK-DLP1 signaling, Gan et al. reported a protective effect of DLP1 inhibitor mdivi-1 on maintenance of normal mitochondrial structure and function in AD cybrid cell [121]. Consistently, two groups demonstrated the rescue effects of DLP1 inhibitor midivi-1 on mitochondria morphology and movements at early stage in APP transgenic mice which alleviated amyloid pathology likely through reducing Aβ production and improved cognitive deficits [109, 122]. The DLP1 inhibitor midivi-1 presumably prevents DLP1 assembly and inhibits DLP1-dependent mitochondrial fission [123]. However, one recent study challenged the specificity of mdivi-1 on mitochondrial fission inhibition and suggested that mdivi-1 might perform as a reversible mitochondrial complex I inhibitor rather than as a specific DLP1 inhibitor [124]. It is important to note that the reported non-DLP1 dependent effects of mdivi-1 were only noticeable when cells received relative high doses of the inhibitor in vitro [124]. Previous in vitro studies did not apply such high concentration of mdivi-1 in neuronal cultures and conclusions should remain valid. However, due to the difficulty in manipulating chemical concentration in in vivo system such as mouse brain, the underlying mechanism of the protective effects of midivi-1 in AD models in vivo thus need to be interpreted carefully [125]. Therefore, more specific methods to inhibit fission or enhance fusion are needed in AD research. In this regard, using real-time PCR and western blot, Manczak et al. explored the protective effect of reduced DLP1 expression in APP mice and found that DLP1 haplodifficiency leads to the restoration of the expression of proteins related to the mitochondrial dynamics, mitochondrial biogenesis and synapses and rescued mitochondrial function in APP transgenic mice as compared to APP transgenic mice alone (Tg2576 line) [126]. Unfortunately, it had not been determined whether DLP1 haploinsufficiency has any beneficial effects on cognitive function and pathological changes in this study. Given that DLP1 knockdown depletes mitochondria from neuronal process and causes synaptic deficits [127, 128], it may have unwanted effects. Perhaps a better approach to correct mitochondrial dynamics abnormalities in AD models is to enhance mitochondrial fusion in vivo instead [129].

### Mitochondrial axonal trafficking deficits and abnormal mitochondrial distribution in AD

In addition to mitochondrial morphological abnormality, mitochondrial distribution was also disturbed in AD brain: mitochondria become less abundant in the neuronal processes in the susceptible pyramidal neurons in AD [101]. This uneven mitochondria distribution in the processes leaves large axonal or dendritic segments devoid of mitochondria, as clearly demonstrated by a recent electron microscopy study [130]. Kinesin-based anterograde transport of mitochondria populates axons with fresh mitochondria, and dynein-based retrograde transport of mitochondria facilitates the recycling of damaged mitochondria and maintains a healthy mitochondrial population in the processes [131, 132]. Therefore, disruption of either anterograde or retrograde transport or both of these processes, either due to faulty mitochondria or impaired mitochondrial transport system, leads to decreased proportion of healthy mitochondria or increased proportion of damaged mitochondria that impaired the integrity and function of mitochondria. It also could significantly affect mitochondrial distribution, which have profound impacts on synaptic and neuronal function [133]. Abnormal changes in mitochondrial transport in AD are under intensive studies.

Mutations in presenilin 1 impair kinesin-based axonal transport through GSK3β activation, which phosphorylates kinesin light chain and releases kinesin from the cargo at sites of membrane insertion [134]. Primary neurons isolated from APP transgenic mice also demonstrated impaired axonal transport of mitochondria [135], which is likely caused by Aβ. Indeed, overexpression of Aβ42 caused mitochondria mislocalization with reduction in axons and dendrites and accumulation in the soma, which contribute to Aβ42-induced neuronal dysfunction in a transgenic Drosophila model in vivo, and this is exacerbated by genetic reductions in mitochondrial transport [115]. Similarly, exposure of neuronal cultures to Aβ oligomers reduces motile mitochondria in axons using live imaging [136–138]. A recent study suggested that amyloid peptides with higher propensity to aggregate also inhibit mitochondrial trafficking [135].

How may Aβ affect the axonal transport of mitochondria? The finding that Cyclophilin D deficiency rescues Aβ-induced axonal mitochondrial transport deficit [139] implicated the potential involvement of calcium and its downstream signaling. Calcium elevation could modulate mitochondrial transport by directly impacting the adaptor proteins involved in mitochondrial transport such as calcium-sensing protein Miro1 or by influencing downstream calcium signaling molecules such as calcineurin and GSK3β [140]. The rescuing effect of mitochondria-targeted antioxidant peptide SS31 on Aβ-induced impaired anterograde axonal transport of mitochondria suggests that oxidative stress could be involved [135]. Motor proteins in both anterograde and retrograde transport can be impacted: Aβ caused reduced expression of anterograde motor proteins KIF5A and restoration of KIF5A corrects Aβ-induced impaired anterograde transport of mitochondria [141]. On the retrograde transport side, oligomeric Aβ interacts with dynein intermediate chain and disrupts the coupling of dynein-Snapin which could potentially impact mitochondrial transport [142]. Microtubule tracks could also be impacted, as Kim et al. suggested that the HDAC6-dependent regulation of α-tubulin acetylation status was essential for Aβ-induced impairment of mitochondrial transport in hippocampus neuronal cultures [143]. Their follow-up study further identified peroxiredoxin1 as another substrate of HDAC6, which is involved in Aβ-induced disruption of ROS, calcium homeostasis and axonal transport in 5xFAD AD model mice and AD patients [144]. Aβ may also impact mitochondrial transport through changes in mitochondrial dynamics through reduction of DLP1 or Mfn2 since reduced DLP1 or Mfn2 cause reduced mitochondrial distribution in the processes [101] and Mfn2 interacts with Miro/Milton complex and is required for axonal transport of mitochondria [145].

Overexpression and/or phosphorylation of tau is another negative regulator for mitochondrial movement in neurons. Earlier studies demonstrated that tau controls the balance of axonal transport through locally differential modulation of dynein and kinesin motor proteins [146], predicting tau accumulation in the somatodendritic compartments compromise axonal anterograde transport. Indeed, tau overexpression preferentially impairs kinesin-dependent anterograde axonal transport of mitochondria and other vesicles through enhanced microtubule binding [147]. Perhaps more relevant to conditions in AD, Shahpasand et al. found that tau phosphorylated at the AT8 sites inhibited mitochondrial movement in the neurite processes of PC12 cells as well as the axons in mouse cortical neurons due to impaired microtubule spacing [148]. Consistent with a critical role in tau phosphorylation in the regulation of mitochondrial transport, neurons from a tau P301L mutant knock-in mouse model had reduced levels of phosphorylated tau but displayed increased anterograde mitochondrial transport in axons [149]. As a result, mitochondrial distribution is progressively disrupted with age in rTg4510 brain and in Alz50-positive neurons in AD brain [150] which probably contributes to significant mitochondrial loss in the tau positive neurons in AD [151]. Interestingly, the effects of Aβ species on mitochondrial movement was subject to the presence of tau proteins. Quintanilla et al. demonstrated that Aβ treatment combined with expression of truncated tau significantly increases the stationary mitochondrial population and the levels of oxidative stress in cortical neurons [152]. Consistently, tau reduction prevented Aβ-induced deficits in the anterograde axonal transport of mitochondria in primary neurons by blocking the activation of GSK3β [153].

Despite the consensual view that Aβ and tau alterations impaired mitochondrial transport, it is unclear whether they specifically impaired mitochondrial transport or also affected other organelles. There is also debate on whether Aβ preferentially impacted anterograde axonal transport of mitochondria or retrograde axonal transport of mitochondria or both [136, 137, 141, 142, 154]. The accumulation of damaged mitochondria at synapses could be the consequence of an impaired retrograde axonal transport of mitochondria [137]. Furthermore, changes in other aspects such as mitochondrial docking that may impact mitochondrial distribution have not been studied [155].

### Impaired mitochondrial biogenesis in AD

There are more than 1000 proteins in neuronal mitochondria, 13 of which are encoded by mitochondrial genome and are hydrophobic proteins that form the core parts of the oxidative phosphorylation complexes of the inner membrane of mitochondria, while the remainder are encoded by nuclear genome [156]. Therefore, mitochondria biogenesis involves coordinated expression between both nuclear and mitochondrial genomes. PGC-1α is considered the master regulator of mitochondrial biogenesis and coordinates/regulates energy metabolism and respiration through interactions with different transcription factors, including nuclear respiratory factor 1 (NRF 1) and nuclear respiratory factor 2 (NRF 2) [157]. NRF-1/2 controls the expression of many nuclear-encoded mitochondrial proteins including mitochondrial transcription factor A (TFAM) which drives the transcription and replication of mtDNA [157]. A complex and multifaceted ROS defense system is linked by PGC-1α to mitochondrial oxidative metabolism, enabling cells to maintain normal redox status in response to changing oxidative capacity [158]. Obviously, mitochondrial biogenesis plays a critical role in maintaining mitochondrial homeostasis during the life cycle of mitochondria.

As discussed earlier, multiple studies demonstrated reduced levels of critical components of the electron transport chain in the brain tissues of AD, which not only underlies the well-documented energy hypometabolism in AD, but may also suggest impaired mitochondrial biogenesis or enhanced mitochondrial clearance. However, mitochondrial clearance through mitophagy is actually impaired in AD (discussed in more detail later). Therefore, an impaired mitochondrial biogenesis is implicated. PGC-1α is abundantly expressed in tissues with high energy demand including the brain. Qin et al. first demonstrated the reduced expression of PGC-1α in AD patients and transgenic mouse model of AD [159]. mtDNA copy numbers were significantly reduced and mitochondrial biogenesis transcriptome signaling is disrupted in laser-capture microdissected pyramidal neurons from AD hippocampus compared to that of control hippocampus [160]. Decreased PGC-1α levels are also associated with abnormal brain insulin signaling, providing one possible mechanism for obesity being a risk factor for AD [161]. Sheng et al. demonstrated that expression of APP Swedish mutant caused reduced expression of PGC-1α and impaired mitochondrial biogenesis likely through a PKA-dependent pathway and restored PGC-1α expression rescued mitochondrial and neuronal functions in cell models of AD [162]. Interestingly, Presenilin 1 is also involved in the regulation of PGC-1α expression through the production of APP intracellular domain (AICD) peptide, the APP processing product after γ-secretase cleavage, and PS1-FAD mutations lost the ability to enhance PGC-1α mRNA levels due to the impaired cleavage of APP proteins in AD [163]. Reciprocally, exogenous expression of PGC-1α in N2a neuroblastoma cells could regulate APP processing by downregulating the transcription of BACE1, which effected decreased secreted Aβ and increased non-amyloidogenic soluble APPα [164]. Another study also suggested that PGC-1α reciprocally regulated BACE1 in vitro and in vivo, in collaboration with SIRT1-mediated deacetylation of PPARγ constituting essential mechanisms for regulation Aβ production in AD [165].

Considering the essential role of PGC-1α impairment on mitochondrial dysfunction and Aβ production in AD, it was attractive to investigate how restoration of PGC-1α expression or its activity would affect mitochondrial and neuronal functions in models of AD. Katsouri et al. studied the potential therapeutic effect of PGC-1α by generating a lentiviral vector to express human PGC-1α in hippocampus and cortex of APP23 transgenic mice [166] and found this abrogated neuronal loss and Aβ aggregation likely through inhibition of BACE-1. In contrast, Dumont et al. crossed the Tg19959 mouse model of AD with transgenic mice overexpressing human PGC-1α protein [167], which unexpectedly exacerbated amyloid and tau accumulation accompanied by an impairment of proteasome activity. The discrepancy of these transgenic animal studies underscores the need to manipulate the expression levels of exogenous PGC-1α proteins in vivo with care because abnormal PGC-1α levels induced toxic effects in some peripheral organs such as in the heart [168].

Another strategy to restore mitochondrial biogenesis was to enhance PGC-1α activity by chemical stimulation [169]. The first evidence came from a study by Dumont et al. in which administration of the PGC-1α agonist bezafibrate exerted neuroprotective effects in a mouse model of tauopathy, as shown by decreased tau pathology and behavioral improvement, which suggested beneficial effect by increased activity in a non-APP model of AD [170]. In an Aβ toxicity mouse model, Gong et al. demonstrated that nicotinamide adenine dinucleotide (NAD+) promoted PGC-1α expression coinciding with enhanced degradation of BACE1 and the reduction of Aβ production in Tg2576 mice in the brain [171]. Furthermore, supplementation of melatonin in drinking water, which enhanced PGC-1α activity in vivo, increased mitochondrial biogenesis and alleviated mitochondrial impairment which led to improved spatial learning and memory deficits, and reduced Aβ deposition and soluble Aβ levels [172].

An alternative strategy is to focus on mitochondrial biogenesis effectors downstream of PGC-1α. Oka et al. examined the effects of human mitochondrial transcriptional factor A (hTFAM) on the pathology of a mouse model of AD (3xTg-AD). They found that expression of hTFAM significantly improved cognitive function, reduced oxidative stress and intracellular Aβ in 3xTg-AD mice and increased expression of transthyretin, known to inhibit Aβ aggregation [173].

Given that 99% of mitochondrial proteins are encoded by nuclear genome and must be imported into mitochondria, mitochondrial biogenesis is heavily dependent on proper mitochondrial protein import [174, 175], which is regulated by protein import machinery in the mitochondrial outer and inner membrane. The translocase of the outer membrane consisting of a pore-forming protein TOM44 and three receptor proteins on the cytosolic side (i.e., TOM20, TOM22, and TOM70) is the main entry gate [175]. Interestingly, Alan Roses reported an association of a polymorphic poly-T variant, rs10524523, in the TOMM40 gene with the age of onset of late-onset AD [176]. While this finding remains controversial [177], it provided the first hint of a possible involvement of mitochondrial import alteration in AD pathogenesis. Indeed, mitochondrial protein import is inhibited by oxidative stress, suggesting that mitochondrial import could be impacted in AD where extensive oxidative damage was documented in susceptible neurons in AD [178]. Gene set enrichment analysis of datasets from patients with AD archived in GeneNetwork showed disruption of mitochondrial import pathway as a hallmark of AD [40]. This was confirmed by investigation of protein expression in the brain tissue which revealed reduction of Tom20 and Tom70, as well as components of OXPHOS complex I and III in AD hippocampus [179].

Several groups had pursued the potential role of APP or Aβ on mitochondrial import machinery [180–184]. Anandatheerthavarada first identified mitochondrial-targeting signal in APP proteins and demonstrated mitochondria APP in cortical neuronal culture and in select regions of the brain of a transgenic mouse model for AD [183]. The follow-up study demonstrated APP is incompletely translocated to mitochondria and forms stable complex with mitochondrial outer and inner membrane translocase in AD brain, which likely blocked mitochondrial import machinery and caused mitochondrial dysfunction [180]. Similarly, Hansson et al. showed that Aβ is translocated to mitochondria through interaction with TOM import machinery and localized to mitochondrial cristae [181]. Aβ also impaired the import competence of mitochondrial precursor proteins although through an extramitochondrial coaggregation mechanism with the inhibitory potency positively correlating with the amyloidogenic capacity [181, 184]. Accumulation of mitochondrial Aβ correlates with early synaptic deficits in AD mouse models [137, 185].

### Abnormal endoplasmic reticulum-mitochondrial interaction in AD

Both endoplasmic reticulum (ER) and mitochondria are continuous tubular networks of membranes in the cytoplasm. Approximately 5–20% of the mitochondrial surface is closely apposed at 10–30 nm distance to ER membrane and form a specialized structure called ER-mitochondria contact sites which provide a stable platform to synergize the function of these two organelles [186, 187]. An expanding number of crucial physiological functions has been ascribed to ER-mitochondria contact sites which include regulation of phospholipid synthesis and metabolism, calcium exchange between ER and mitochondria, regulation of mitochondrial dynamics and autophagy, inflammasome activation, and apoptosis [187–189], all of which are essential for proper mitochondrial function. Emerging evidence demonstrated a crucial role of ER-mitochondria contact sites in neuronal function and survival and disturbed MAM signaling and function is increasingly implicated in neurodegenerative diseases including AD [190].

The potential involvement of MAM dysfunction in AD was first implicated by Eric Schon’s finding of the MAM localization of presenilin 1 and 2 as well as the gamma-secretase activity [191, 192]. Later studies demonstrated that APP and β-secretases are also present and harbor APP processing activities in MAMs [193], this is consistent with the notion that APP processing occurs at lipid raft domains which is present in MAMs. Indeed, a considerable amount of Aβ was produced at mitochondria-ER contact sites in wild type mouse brain [194] which makes ER-mitochondria contact sites a likely focal point for toxic effects of Aβ. Importantly, Hedskog et al. found up-regulated MAM-associated proteins in the AD brain, and demonstrated dysregulated MAM occurs during the course of disease in a transgenic AD mouse model [195], although direct evidence of specific alterations in the ER-mitochondria contact sites in AD patient are still lacking. Mutations in the *C. elegans* gene encoding a PSEN homolog, *sel-12* resulted in elevated endoplasmic reticulum (ER)-mitochondrial Ca^2+^ signaling and an increase in mitochondrial superoxide production [196]. Molecular changes of MAM components occurred in the cerebral cortex of 3 months old APP/PS1 mice assayed using label-free LC-MS/MS which suggest that MAM dysregulation is likely an early event in vivo [197].

Consistent with the in vivo findings, multiple groups demonstrated aberrantly increased ER-mitochondria contacts and/or enhanced MAM function in various cell models of AD which enables more detailed mechanistic studies: for example, overexpression of APP mutants or exposure to nanomolar concentrations of Aβ increases ER-mitochondria contact points and mitochondrial calcium concentrations [193, 195]. Enhanced cholesteryl ester and phospholipid synthesis were found in presenilin-1 and -2 double knockout mouse embryonic fibroblast cells and in fibroblasts from patients with both the familial and sporadic forms of AD, suggesting an aberrant upregulation of MAM function and ER-mitochondria crosstalk in these cell models [198]. Follow-up studies from this same group demonstrated that presenilins likely regulates ER-connectivity and function through APP processing since accumulation of C99, the 99-aa C-terminal fragment of APP after beta-secretase cleavage and a substrate of gamma-secretase, at MAM caused elevated sphingolipid turnover and increased ceramide which altered lipid composition and thus impacted the ER-mitochondria contacts, resulted in metabolic disturbance and reduced mitochondrial respiration [199]. Consistent with an enhanced ER-mitochondria connectivity and function, increased C99 levels and ceramide levels were found in MAM fractions in cell and animal models of AD and in fibroblasts from AD patients carrying PS2 mutations [199]. Neuronal MAM was also subject to the regulation of extraneuronal apolipoprotein that is associated with increased AD risk. Tambini et al. suggested apolipoprotein E (ApoE4) secreted by astrocytes significantly increased ER-mitochondrial communication and MAM function as measured by the synthesis of phospholipids and of cholesteryl esters [200]. Consistently, disrupted cholesterol homeostasis and related neurotoxicity in AD were shown to be mediated by ER-mitochondria stress triggered by Aβ that promoted cholesterol synthesis and mitochondrial cholesterol influx [201].

However, there is controversy on the effects of presenilins on MAM structure and function since in tissues from AD patients carrying PS1 E280A mutation, ER-mitochondria tethering was impaired, a result further confirmed by in vitro studies [202]. Pizzo’s group also reported that PS2 expression, but not its ablation, enhanced both physical interaction and function coupling of ER-mitochondria likely through modulation of Mfn2 antagonism of ER-mitochondria interactions [203]. While further studies are needed to resolve the discrepancy, it should be noted that both enhanced and disturbed ER-mitochondria tethering could lead to ER and mitochondrial dysfunction and cause mitochondrial dysfunction. In fact, it is not without precedence that both enhanced and disturbed ER-mitochondria tethering could contribute to the same neurodegenerative disease as in the case of Parkinson’s disease where Parkin mutations enhanced [204], but alpha synuclein and DJ-1 mutations disturbed [205, 206], ER-mitochondrial tethering. Overall, these studies collectively demonstrated that abnormalities in ER-mitochondria tethering contributes to mitochondrial dysfunction in AD. Now, it would be of importance to understand whether and how such abnormalities in ER-mitochondria tethering relates to other pathological changes such as impaired mitophagy and inflammasome activation in AD.

### Impaired mitophagy in AD

As metabolic active organelles where more than 90% of reactive oxygen species (ROS) are produced [57], mitochondria develop a sophisticated mitochondrial quality control system to cope with unavoidable damage to its contents as well as the organelles as a whole. At the organelle level, damaged mitochondria are degraded through mitophagy. The most well characterized mitophagy pathway involves stabilization/activation of PINK1 at the outer mitochondrial membrane by impaired mitochondrial membrane potential characterizing damaged mitochondria [207]. PINK1 not only phosphorylates and recruits the E3-ubiquitin ligase, Parkin, to mitochondria, but also phosphorylates ubiquitin to feed Parkin mediated ubiquitination of mitochondrial outer membrane proteins which labels the damaged mitochondria for degradation through mitophagy pathway [208]. It is of importance to note that mutations in either PINK1 or PARKIN are associated with early-onset familial Parkinson disease, the second most common neurodegenerative disease after AD [209], suggesting the critical role of this pathway in the CNS.

Mitochondria are key targets of autophagic degradation in the brain of AD patients [210, 211] and strong evidence suggests autophagy/lysosome failure in AD [212]. Accumulation of damaged mitochondria as evidenced by swollen appearance with distorted cristae have been identified by electron microscopy studies both in biopsy of human AD cases and in transgenic animal models of AD [87, 109, 213]. Increased PINK1, Parkin and/or increased ubiquitination of mitochondrial proteins were found in the accumulated mitochondria in pyramidal neurons in AD hippocampus, APP transgenic mice and cell models expressing mutant APP or PS1 or isolated from human AD patients, implicating an activated but stalled mitophagy process [213–215]. It is likely that inadequate mitophagic capacity in eliminating increased number of damaged mitochondria [214] or impairment in the later steps in mitophagy involving lysosomal degradation [40, 213, 216, 217] that resulted in the accumulation of damaged mitochondria and disturbance in mitochondrial homeostasis.

Mechanistically, PS1 promotes PINK1 promoter transactivation, mRNA and protein expression through AICD, the cleavage product of APP by gamma-secretase yet PS1 mutations disrupted lysosomal acidification and proteolysis due to the failure of v-ATPase targeting to lysosomes during autophagy [218]. Corsetti et al. described enhanced mitophagy triggered by a specific form of tau protein in vitro AD model. They reported a 20–22 kDa NH2-tau fragment that contributed to synaptic deterioration in AD by aberrantly recruiting Parkin and UCHL-1 to mitochondria which made them more prone to detrimental autophagic clearance [219]. However, Hu et al. found increased tau protein might increase mitochondrial membrane potential that prevents mitochondrial recruitment of Parkin by PINK1 in AD [220]. A most recent study found significantly less mitophagy events in AD hippocampus and in human neurons generated from iPS cells from AD patients bearing APP mutation or two copies of ApoE4 and pinpoint the impaired orchestration of mitophagy at earlier steps of initiation in AD due to decreased levels of activated mitophagic proteins, such as p-TBK1 and p-ULK1 [215].

While detailed mechanisms underlying impaired mitophagy in AD remains to be worked out, enhancing mitophagy by either genetic manipulation or pharmaceutical methods appears beneficial across different AD models. Overexpression of PARK2 reversed mitophagy failure and led to the recovery of mitochondrial membrane potential in sAD fibroblasts [213]. Overexpression of PINK1, by activating mitophagy signaling in APP transgenic mice, restores mitochondrial function, reduces Aβ production and amyloid pathology, and alleviates synaptic function as well as cognitive/behavioral functions [221]. Treatment with actinonin or urolithin A, compounds that enhance mitophagy, restored normal memory to *C. elegans* models overexpressing _Aβ1–42_ or tau in a PINK1-dependent manner [215]. Importantly, these compounds similarly enhanced mitophagy and restored normal mitochondria in APP/PS1 mice, which resulted in alleviation of amyloid pathology and improved cognitive/behavioral functions. Interestingly, the clearance of amyloid plaques appears due to increased phagocytic efficiency of microglia whose mitophagy is also impaired in APP/PS1 mice and enhanced by these treatments. This underscores an intertwined role of defective mitophagy in the development of amyloid pathology, which makes mitophagy as an important node for intervention. Recent studies demonstrated enhanced mitophagy protects against inflammasome-mediated neuroinflammation, a prominent feature of AD, which makes it a more promising target [222]. In this regard, NAD^+^-boosting compounds such as nicotinamide riboside (NR) potently induce mitophagy [223, 224]. NR treatment restored mitochondrial function and robustly decreased amyloid pathology and improved context-dependent memory in the APP/PS1 mice [40].

### Impaired mitochondrial proteostasis in AD

At protein level of mitochondrial quality control mechanisms, a process called mitochondria proteostasis monitors mitochondrial protein damages through an interconnected network consisting of chaperones and proteases in each compartment of mitochondria: Mitochondrial chaperones are involved in protein translocation and folding reactions while ATP-dependent proteases are responsible for directly removing damaged or misfolded proteins from mitochondria [225–229]. Defects in these proteins impair the capability of mitochondria to monitor, repair and remove damaged proteins which eventually induces accumulation of protein aggregation within mitochondria and causes mitochondrial dysfunction [230–233]. Importantly, genetic mutations in all these mitochondrial chaperones and proteases cause human diseases with severe neurological symptoms [229, 234–238] which underscores the significance of mitochondrial proteostasis regulated by mitochondrial protease and chaperones in mitochondrial function/dysfunction in the nervous system.

At the suborganelle level, abundant evidence demonstrated accumulation of damaged mitochondrial contents including mtDNA and proteins in AD [239], which suggest mitochondrial proteostasis may also be impaired. In general, maturation of imported mitochondrial proteins and degradation of damaged proteins remain in balance. However, one study actually found mitochondrial proteases and chaperones are upregulated in AD patients [50] possibly as an insufficient protective response. Upregulation of these mitochondrial proteases and chaperones in the brain of MCI patients and in the 3XTgAD mice precedes amyloid and tau pathology, suggestive of an early event during the course of disease [40]. Moreover, enhanced mitochondrial proteostasis might reduce Aβ proteotoxicity in AD animal models [40].

Current studies focused on two mitochondrial proteases related to the processing and metabolism of APP or Aβ. One is the mitochondrial peptidasome, PreP, located in mitochondria matrix which is involved in the cleavage/maturation of presequence of mitochondrial matrix proteins after its import [182]. Falkevall et al. first identified that the PreP was capable of degrading Aβ40 and Aβ42 in vitro, revealing the likely Aβ degradation mechanism in mitochondria [240]. Consistent with this biochemical study, Alikhani et al. reported decreased activity of PreP in AD patients and transgenic AD mice in which increased oxidative stress likely underlies the decreased PreP activity in AD [241]. In addition to detrimental effects of oxidative stress on PreP activity, one study suggested a feedback mechanism of Aβ on PreP activity that triggered imbalanced mitochondrial proteome due to the rapid degradation of impaired preprotein maturation [242]. These studies were further corroborated in vivo utilizing PreP transgenic mice. In PreP-overexpressed AD transgenic mice, increased expression of human PreP in cortical neurons attenuated mitochondrial amyloid pathology and synaptic mitochondrial dysfunction [243]. It is interesting to note that genetic mutations in PreP associated with an autosomal recessive, slowly progressive syndrome characterized by mental retardation, spinocerebellar ataxia, cognitive decline and psychosis [244]. Although it was impractical to analyze the pathological changes in the brains of these patients in this study, PreP (+/−) heterozygous mouse showed progressive ataxia associated with brain degenerative lesions, including accumulation of Aβ-positive amyloid deposits thus providing a mechanistic demonstration of the mitochondrial involvement in amyloidotic neurodegeneration.

The other mitochondrial protease of more interest in AD field is HtrA2/Omi, a serine protease in the mitochondrial intermembrane space (IMS). A weak association between HtrA2 A141S and AD was found in Swedish case control studies and specific protease activity of HtrA2 was found to be significantly increased in AD patients [245]. Using yeast-two hybrid assay, it was found that HtrA2/Omi interacts with Aβ through the PDZ domain at C-terminus, which was further confirmed in HEK392 cells by co-immunoprecipitation assay [246]. Aβ was found in matrix, but there is no consensus on its localization in mitochondrial compartments. It is possible that Aβ may have access to HtrA2 in the intermembrane space, but how such interaction impacts HtrA2 activity is unknown. A later study demonstrated that HtrA2/Omi performs a chaperone function and significantly delays the aggregation of Aβ_1–42_ peptide but independent of the PDZ domain in vitro [247]. Densely accumulated HtrA2 immunoreactivity was identified extracellularly in the cortex and hippocampus of AD patients [248], suggesting changes in HtrA2 may affect amyloid deposition extracellularly which is consistent with the partial localization of HtrA2 immunoreactivity in amyloid plaques. However, the physiological significance of these observations in vivo as it relates to mitochondria is unclear. APP is localized to mitochondria and partially colocalizes with HtrA2. Interestingly, mitochondrial APP is directly and efficiently cleaved by the HtrA2 both in vitro and in vivo, which releases C161 fragments into cytosol [249]. It is postulated that HtrA2 cleavage of APP may alleviate APP accumulation induced mitochondrial dysfunction. However, the fate of C161 related to amyloidogenic- and non- amyloidogenic pathways were not determined. Adding more complexity to the role of HtrA2, it also interacts with presenilin in active γ-secretase complexes located to mitochondria and modulates the cleavage of APP [250]. Interestingly, such interaction also impacts the HtrA2 protease activity since the C-terminus of PS1 is an active peptide ligand for the PDZ domain of HtrA2 and induces HtrA2-dependent cell death [251].

Overall, despite ample evidence demonstrating accumulation of damage mitochondrial proteins, there were sparse studies on alterations in mitochondrial proteostasis in AD. Whether and how specific mitochondrial chaperones and proteases in different mitochondrial compartments are involved is not clear. Importantly, recent studies demonstrated “mitochondria as guardian in cytosol” where mitochondria proteases degrade aggregation-prone cytosolic proteins after their importation [252, 253], suggesting that mitochondrial proteostasis could also regulate cytosolic protein homeostasis and neuronal integrity. However, this important aspect remains to be explored in AD.

## Conclusion

Mitochondrial dysfunction plays a critical role in AD either as a primary or secondary event, which could represent promising therapeutic targets (Fig. 1). One therapeutic strategy targeting mitochondria directly focuses on modulating mitochondrial activities such as bioenergetics. For example, oxaloacetate (OAA), a Krebs cycle and gluconeogenesis intermediate that enhanced bioenergetic fluxes and upregulated some brain bioenergetic infrastructure-related parameters, is in phase I clinical trial [254]. NAD, an intermediate common to several mitochondrial metabolic pathways such as glycolysis, TCA cycle, and oxidative phosphorylation, is in phase II trial for AD [255]. Interestingly, recent studies demonstrated that mild inhibition of complex I at the FMN subunit (i.e., NDUFA1) by CP2 compound augmented respiratory capacity and reduced proton leaks. CP2 could alleviate cognitive and pathological deficits in various animal models of AD and is also being developed to treat AD [256], suggesting the potential use of bioenergetics modulators in AD treatment. However, it must be noted that partial complex I deficiency induced by neuronal ablation of another complex I subunit, NDUFA5, caused mild chronic encephalopathy in mice [257], suggesting that selection of safe bioenergetics modulators for AD treatment is likely a considerable challenge due to our incomplete understanding of the mechanisms behind these differential outcomes.

Alternatively, given that the loss of mitochondrial structural and functional integrity is likely causally associated with impaired energy metabolism and increased oxidative stress during the course of AD, it probably holds greater promise to consider restoration of the integrity of mitochondria for therapeutic development, which requires better understanding of the underlying mechanisms. Indeed, recent advances in the field demonstrated the involvement of impaired mitochondrial dynamics and transport, biogenesis and mitophagy, proteostasis and quality control as well as its interactions with other organelles such as ER, which offers multiple novel intervention sites to target the integrity of mitochondria. Most of the mechanistic studies rely on genetic models of AD in vitro and in vivo, which demonstrated how APP or Aβ causes impaired mitochondrial integrity and dysfunction by impacting these mitochondrial regulatory mechanisms separately. However, the relationship between these deficits and their relative contribution to APP- or Aβ-induced mitochondrial dysfunction and neuronal dysfunction was not elucidated. It is possible that they act together in a downward spiral manner to impair mitochondrial integrity and cause mitochondrial dysfunction that mediates neurotoxic effect of APP/Aβ. This implies that restoration of one deficit may have an overall beneficial effect on mitochondrial integrity and function as repeatedly demonstrated in these studies.

More studies are needed to address how mitochondria become dysfunctional in the case when mitochondrial dysfunction plays the primary role in the pathogenesis. Because aging is the only known risk factor for this subset of AD, given the multifaceted nature of biological aging, the lack of specific models is the biggest hurdle. Nevertheless, as stipulated by the “mitochondrial cascade hypothesis”, the accumulation of somatic mtDNA mutations with advancing aging influences brain function. Many of these mitochondrial regulation mechanisms become loose during aging. For example, mitochondrial biogenesis and mitophagy decline while mitochondria fission enhances along aging. It is possible that mild deficit in one or several of these mitochondrial regulation mechanisms determined by one’s genetic background could set the motion of a downward spiral and all of these mitochondrial deficits could come into play at some points that impairs mitochondrial integrity, which causes damage, affects repair and/or replication of mtDNA and thus accelerates the accumulation of mtDNA changes, leading to mitochondrial dysfunction and eventually, the disease (Fig. 1). Therefore, the better understanding of how these mitochondrial regulation mechanisms are involved in AD, either as a primary or secondary event, could potentially provide multiple novel therapeutic targets that benefit all AD patients.

## Data Availability

Not applicable.

## Abbreviations

AD: Alzheimer’s disease
Aβ: Amyloid-β
ApoE4: Apolipoprotein E
BACE1: Beta-APP cleaving enzyme
ETC: Electron transfer chain
ER: Endoplasmic reticulum
hTFAM: human mitochondrial transcriptional factor A
MAM: Mitochondria-associated membrane
mtDNA: mitochondrial DNA
TFAM: Mitochondrial transcription factor A
MDVs: Mitochondrial-derived vesicles
NRF 1/2: Nuclear respiratory factor 1 and 2
PGC-1α: Peroxisome proliferator activator receptor gamma-coactivator 1α
ROS: Reactive oxygen species
SOD2: Superoxide dismutase 2
TIM23: Translocase of the inner mitochondrial membrane 23
TOM40: Translocase of the outer mitochondrial membrane 40
